# Novel absorbance peak of gentisic acid following the oxidation reaction

**DOI:** 10.1371/journal.pone.0232263

**Published:** 2020-04-29

**Authors:** Sho Hosokawa, Kenichi Shukuya, Keisuke Sogabe, Yasukazu Ejima, Tatsuya Morinishi, Eiichiro Hirakawa, Hiroyuki Ohsaki, Tatsuo Shimosawa, Yasunori Tokuhara

**Affiliations:** 1 Department of Medical Technology, Ehime Prefectural University of Health Sciences, Ehime, Japan; 2 Department of Medical Technology and Sciences, School of Health Sciences at Fukuoka, International University of Health and Welfare, Fukuoka, Japan; 3 Kaneka Techno Research Corporation, Hyogo, Japan; 4 Kaneka Corporation, Vinyls and Chlor-Alkali Solutions Vehicle, Osaka, Japan; 5 Department of Medical Technology, Kagawa Prefectural University of Health Sciences, Kagawa, Japan; 6 Department of Medical Biophysics, Kobe University Graduate School of Health Sciences, Kobe, Japan; 7 Clinical Laboratory Medicine, School of Medicine, International University of Health and Welfare, Chiba, Japan; Consejo Superior de Investigaciones Cientificas, SPAIN

## Abstract

Gentisic acid (GA), a metabolite of acetylsalicylic acid (ASA), and homogentisic acid (HGA), which is excreted at high levels in alkaptonuria, are divalent phenolic acids with very similar structures. Urine containing HGA is dark brown in color due to its oxidation. We recently reported a new oxidation method of HGA involving the addition of sodium hydroxide (NaOH) with sodium hypochlorite pentahydrate (NaOCl·5H_2_O), which is a strong oxidant. In the present study, we attempted to oxidize GA, which has a similar structure to HGA, using our method. We herein observed color changes in GA solution and analyzed the absorption spectra of GA after the addition of NaOH with NaOCl·5H_2_O. We also examined the oxidation reaction of GA using a liquid chromatography time-of-flight mass spectrometer (LC/TOF-MS). The results obtained indicated that GA solution had a unique absorption spectrum with a peak at approximately 500 nm through an oxidation reaction following the addition of NaOH with NaOCl·5H_2_O. This spectrophotometric method enables GA to be detected in sample solutions without expensive analytical instruments or a complex method.

## Introduction

**Fig 1:**
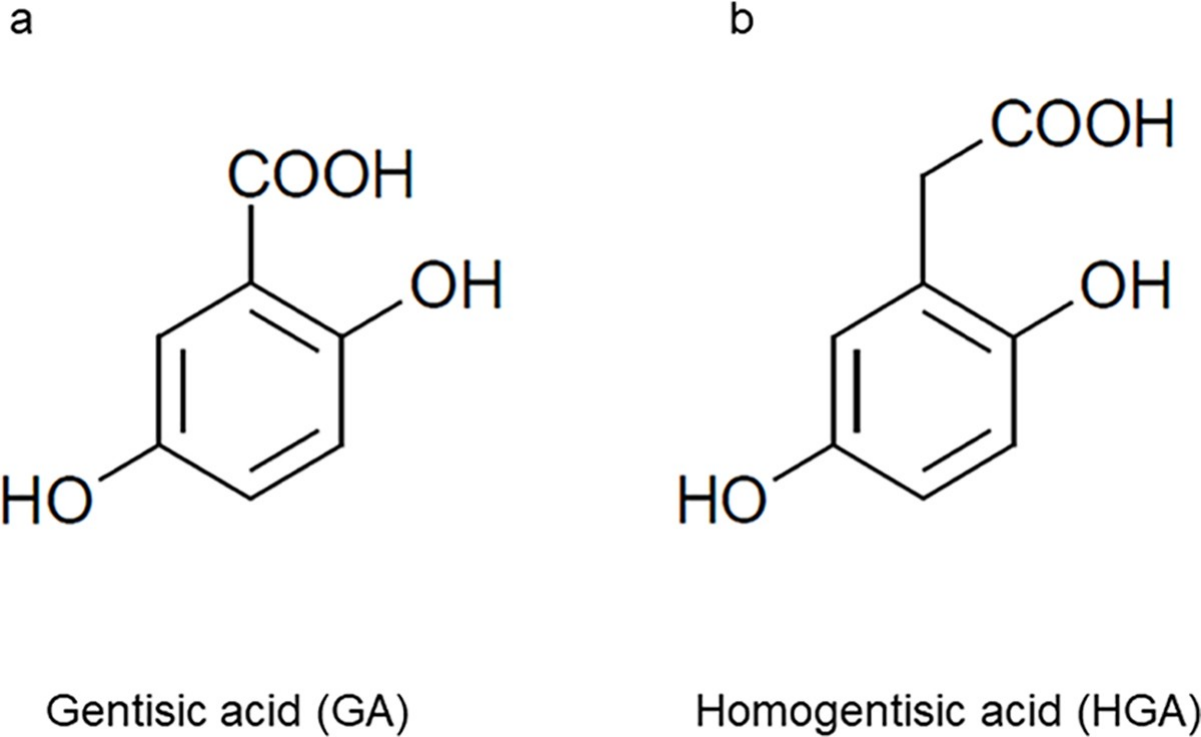
Structures of gentisic acid (GA) and homogentisic acid (HGA). (a) Gentisic acid. (b) Homogentisic acid.

Gentisic acid (2,5-dihydroxybenzoic acid, GA), one of the metabolites of acetylsalicylic acid (ASA), is excreted into the urine in excessive amounts under various conditions [1]. GA is a divalent phenolic acid with a similar structure to homogentisic acid (2,5-dihydroxyphenylacetic acid, HGA) (Fig 1). HGA is excreted at excessive amounts in the urine of patients with alkaptonuria, which is a hereditary metabolic disorder causing the accumulation of HGA. Alkaptonuric urine containing HGA turns dark brown in color when left to stand due to the oxidation of HGA to benzoquinone acetic acid (BQA) [2].

We previously developed a new spectrophotometric detection method for HGA with a chemical property that shows a color change when oxidized to BQA using sodium hypochlorite pentahydrate (NaOCl·5H_2_O), which is a strong oxidant with a solid (finely ground) form, and an effective chlorine concentration of approximately 42% under alkaline conditions [3]. We previously reported that alkaptonuric urine and HGA solution following the addition of sodium hydroxide (NaOH) with NaOCl·5H_2_O rapidly turned dark brown and exhibited characteristic absorption spectra with peaks at 406 and 430 nm, respectively [3, 4]. Therefore, we attempted to oxidize GA, a divalent phenolic acid with a similar structure to HGA (Fig 1), using our spectrophotometric method, which detects a color change in HGA after oxidization following the addition of NaOH with NaOCl·5H_2_O.

In the present study, we observed changes in the absorption spectra of GA following the addition of NaOH with NaOCl·5H_2_O. We also examined color changes in GA solution using liquid chromatography time-of-flight mass spectrometry (LC/TOF-MS). The results obtained indicated that the rapid color change in GA solution reflected the oxidation reaction caused by the addition of a combination of NaOH and NaOCl·5H_2_O and showed characteristic absorption spectra. This new method enables the detection of GA in sample solutions.

## Materials and methods

### Reagents

ASA, ascorbic acid (AA), GA, salicylic acid (SA), and 1 mol/L NaOH were purchased from Wako Pure Chemical Industries, Ltd. (Osaka, Japan). HGA was purchased from Tokyo Chemical Industry Co., Ltd. (Tokyo, Japan). NaOCl·5H_2_O was obtained from Kaneka Co., Ltd. (Osaka, Japan).

### Apparatus

A model U-2900 spectrophotometer (Hitachi High-Technology Co., Ltd., Tokyo, Japan) with microcells with a 10-mm path length was used to measure the absorption spectra of sample solutions. LC/TOF-MS was performed on a Shimadzu Corporation Nexera X2 UHPLC System and Bruker Daltonics maXis 4 G.

### Preparation of samples

A standard stock solution of 1000 mg/L GA was prepared by dissolution in distilled water. The solution was diluted to 900, 800, 700, 600, 500, 400, 350, 300, 250, 200, 190, 180, 170, 160, 150, 140, 130, 120, 110, 100, 90, 80, 70, 60, 50, 40, 30, 20, 10, 9, 8, 7, 6, 5, 4, 3, 2, and 1 mg/L with distilled water. NaOH with NaOCl·5H_2_O solution was prepared by mixing 1mol/L NaOH with NaOCl·5H_2_O at 1:50 (w/w).

### Spectrophotometry

GA samples from 1 to 1000 mg/L were tested in this experiment. A total of 0.8 mL of each sample was added to a quartz cell, followed by 10 μL NaOH with NaOCl·5H_2_O or 10 μL NaOH, and the sample solutions were then mixed well. The solutions were incubated at room temperature for 5 min, and absorption spectra and absorbance values at 500 nm were measured. Similarly, 0.8 mL of ASA (400 mg/L), SA (400 mg/L), or a mixture of GA, ASA, and SA (the final concentration of each was 400 mg/L) solution was incubated with 10 μL of NaOH with NaOCl·5H_2_O for 5 min and absorption spectra were measured. Furturmore, 10 μL of NaOH with NaOCl·5H_2_O was added to 0.8 mL of 400 mg/L GA containing several concentrations of AA (10, 50, 100, 200, and 300 mg/L). Sample solutions were incubated at room temperature for 5 min and absorption spectra were measured. All measurements were performed with a 1-nm bandwidth at a scan speed of 100 nm/min.

### LC/TOF-MS

Ten microliters of NaOH or NaOH with NaOCl·5H_2_O was added to 0.8 mL of 400 mg/L GA solution. Sample solutions were incubated at room temperature for 5 min and introduced directly without passing through the column. The mobile phase was 5% methanol aqueous solution and the flow rate was 0.2 mL/min. The ESI capillary voltage was set at 3 kV, gas temperature at 200°C, and gas flow at 2 L/min. Mass spectra (m/z 50–1500) were acquired in the negative ion mode.

## Results

### Color changes in and absorption spectra of ASA and its metabolites

**Fig 2:**
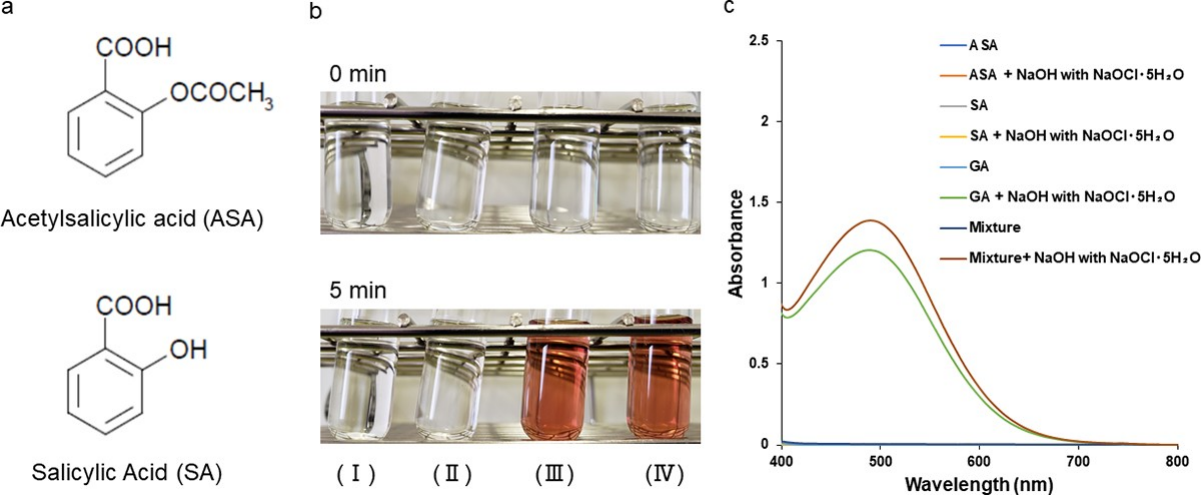
Color changes in and absorption spectra of GA and GA analogs. (a) Structures of acetylsalicylic acid (ASA) and salicylic acid (SA). (b) 400 mg/L ASA (I), 400 mg/L SA (II), 400 mg/L GA (III), and a mixture of GA, ASA, and SA (the final concentration of each was 400 mg/L) (IV) before and after the addition of NaOH with NaOCl·5H_2_O. (c) Absorption spectra of 400 mg/L ASA, 400 mg/L SA, 400 mg/L GA, and the mixture before and after the addition of NaOH with NaOCl·5H_2_O.

GA (Fig 1A) and SA (Fig 2A) are the major metabolites of ASA (Fig 2A). To examine the specificity of the GA peak, we added NaOH with NaOCl·5H_2_O to ASA, SA, GA, or a mixed solution and observed color changes. The ASA and SA solutions were transparent and color changes were not recognized by the naked eye before or after the reaction (Fig 2B). On the other hand, GA and mixture solutions visibly changed in color from transparent to dark brown before and after the reaction (Fig 2B). We then measured their absorption spectrum changes (400–800 nm). Before the addition of NaOH with NaOCl⋅5H_2_O, all sample solutions were clear and colorless and showed almost the same absorption spectra near the baseline (absorbance values between -0.0055 and 0.0201) (Fig 2C). Even after the addition of NaOH with NaOCl·5H_2_O, the ASA and SA solutions did not show any absorption peaks. On the other hand, GA and mixture solutions showed an absorbance peak at approximately 500 nm (Fig 2C).

### Detection range of GA

**Fig 3:**
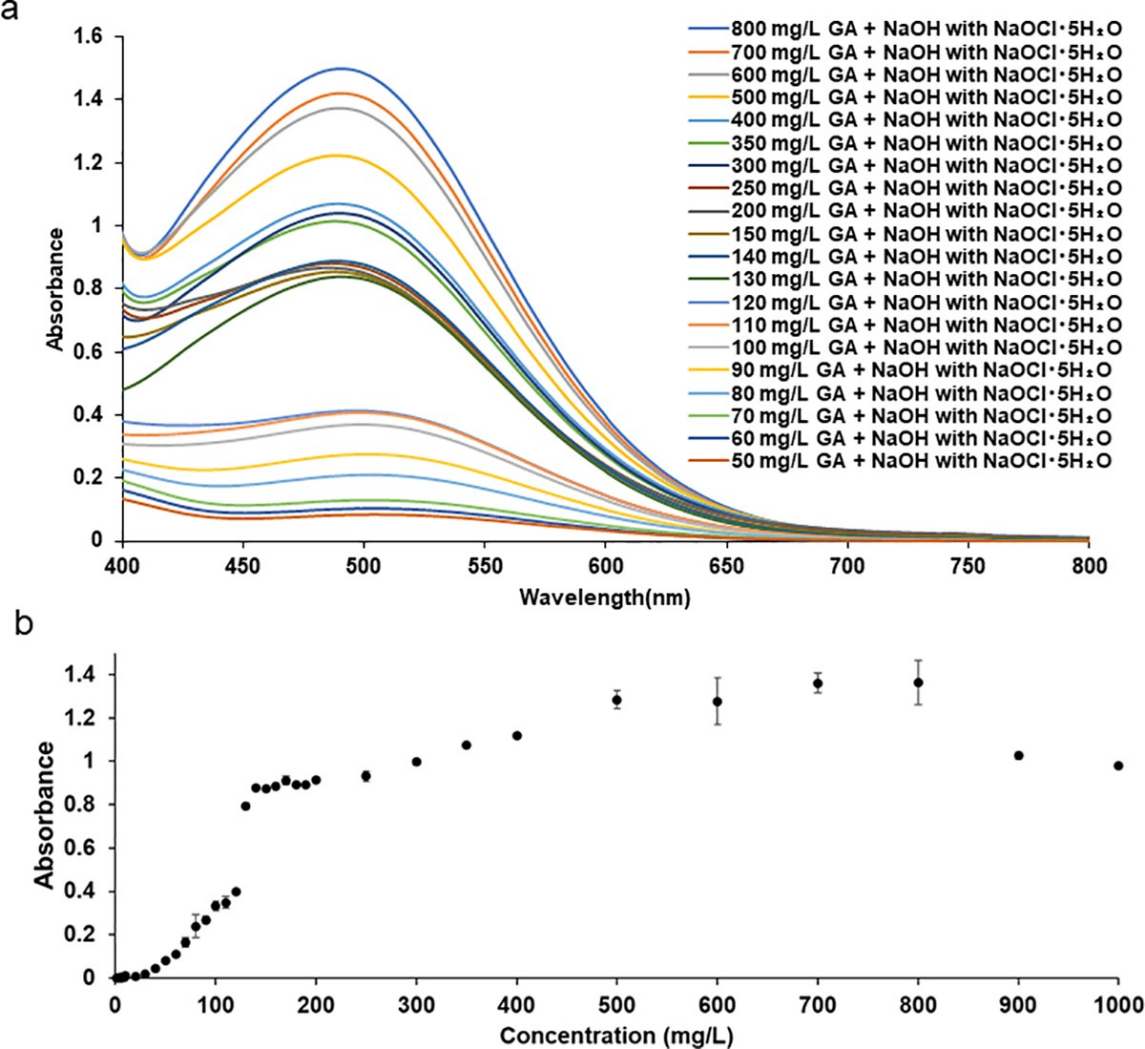
Color changes in and absorption spectra of GA. (a) Absorption spectra of GA from 50 to 800 mg/L after the addition of NaOH with NaOCl·5H_2_O. (b) Absorbance at 500 nm of GA from 1 to 1000 mg/L after the addition of NaOH with NaOCl·5H_2_O. Results are the mean ± S.D. of three experiments.

We added NaOH with NaOCl⋅5H_2_O to GA sample solutions at concentrations ranging between 1 and 1000 mg/L and then measured their absorption spectra (400–800 nm) and absorbance values at 500 nm to investigate the detection range of our spectrophotometric method. GA solutions containing more than 60 mg/L showed an absorbance peak at approximately 500 nm detected by the spectrophotometer after the addition of NaOH with NaOCl·5H_2_O (Fig 3A). We also measured the absorbance values at 500 nm of GA solutions from 1 to 1000 mg/L (Fig 3B).

### Effects of additive reagents

**Fig 4:**
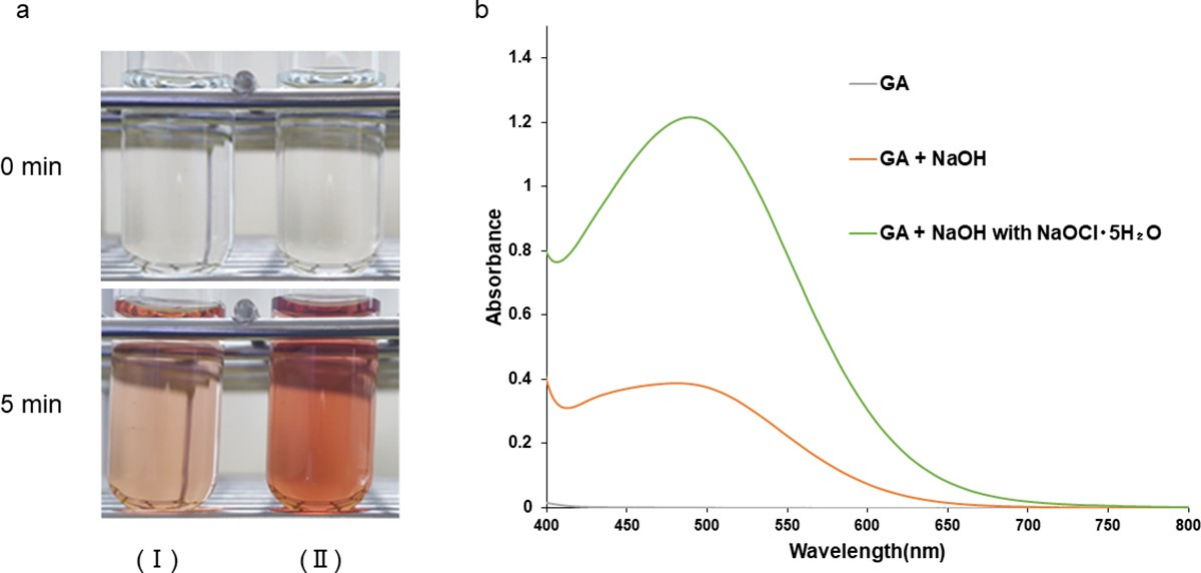
Color changes in and absorption spectra of GA after the addition of different additive reagents. (a) 400 mg/L GA after the addition of NaOH (I) or NaOH with NaOCl·5H_2_O (II). (b) Absorption spectra of 400 mg/L GA after the addition of NaOH or NaOH with NaOCl·5H_2_O.

We added NaOH or NaOH with NaOCl·5H_2_O to GA solution and observed color changes. GA solution became a darker brown color after the addition of NaOH with NaOCl·5H_2_O than with the addition of NaOH (Fig 4A). We then conducted a spectrophotometric analysis in the visible region (400–800 nm). The absorbance curve of the GA solution showed a sharper peak at approximately 500 nm after the addition of NaOH with NaOCl·5H_2_O than after the addition of NaOH (Fig 4B).

### Mass spectrometry

**Fig 5:**
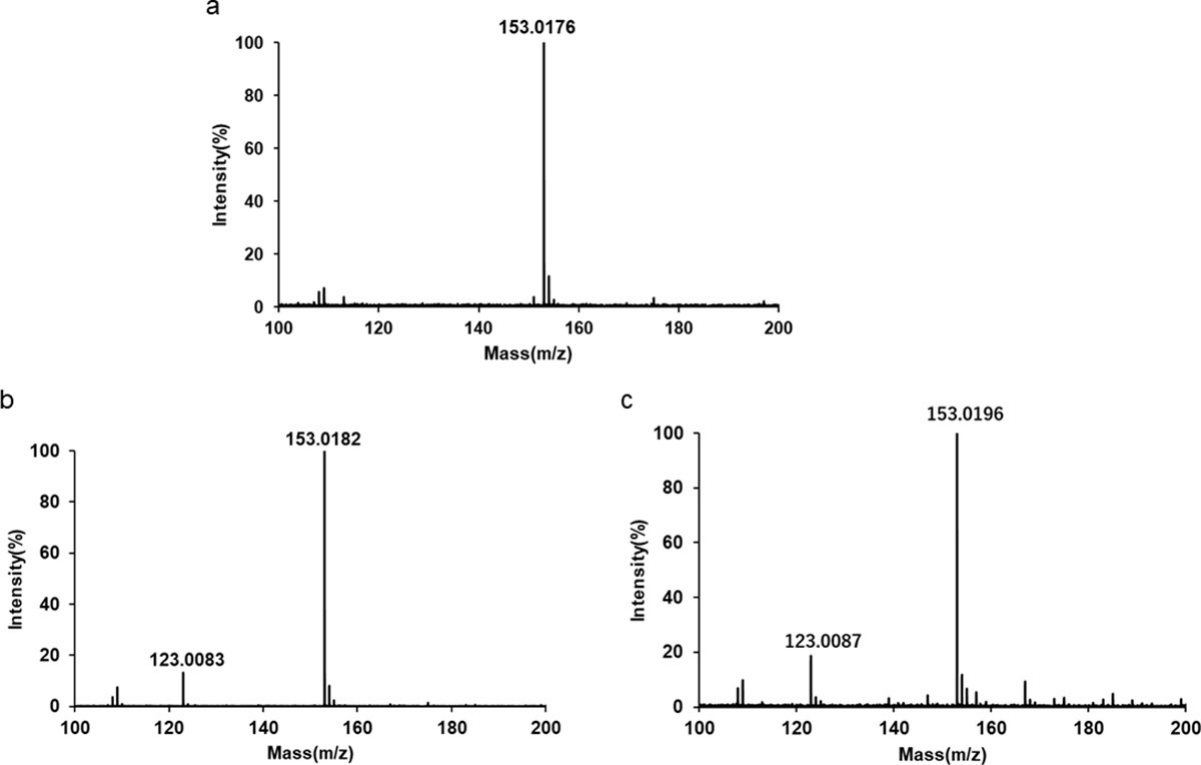
LC/TOF-MS spectra of GA. (a) MS spectrum of 400 mg/L GA. (b) MS spectrum of 400 mg/L GA after the addition of NaOH. (c) MS spectrum of 400 mg/L GA after the addition of NaOH with NaOCl·5H_2_O.

We analyzed the color reaction of GA solution using LC/TOF-MS. GA was represented by a peak at m/z 153 [M-H] ^−^ (Fig 5A). GA solution after the addition of NaOH or NaOH with NaOCl·5H_2_O showed a new peak at m/z 123 (Fig 5B and 5C).

### Effects of ascorbic acid

**Fig 6:**
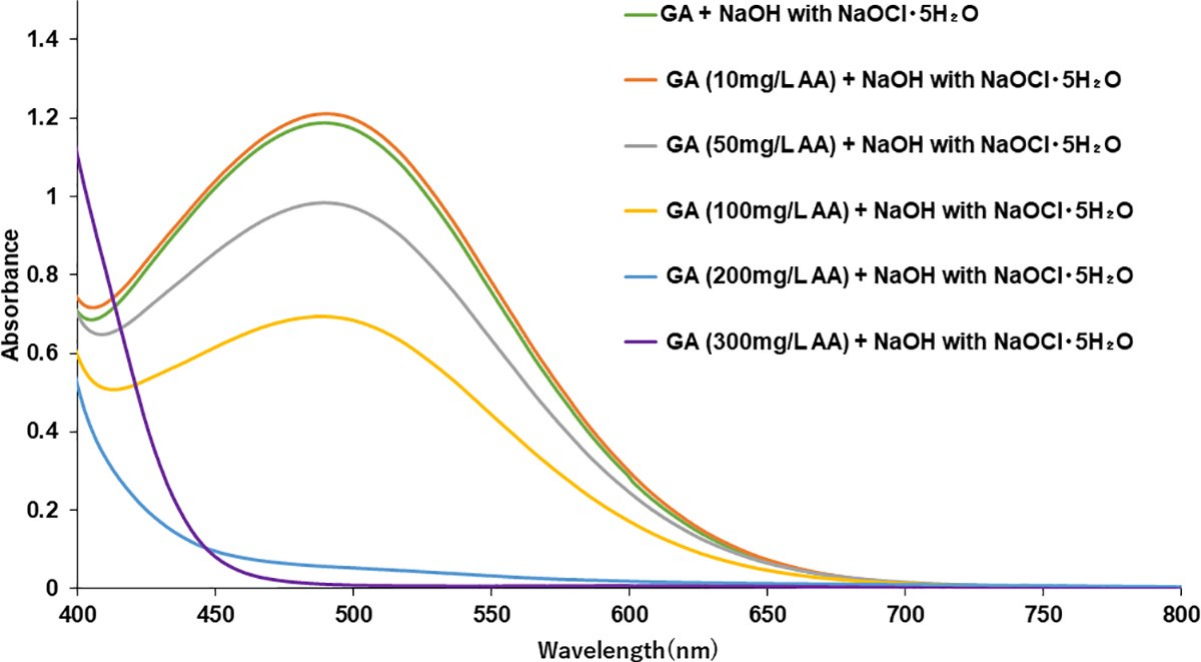
Effects of AA on the absorption spectra of GA after the addition of NaOH with NaOCl·5H_2_O. Absorption spectra of 400 mg/L GA solutions containing several concentrations of AA (10, 50, 100, 200, and 300 mg/L) after the addition of NaOH with NaOCl·5H_2_O.

We investigated whether AA, an antioxidant, affected the absorption spectrum of GA solution. We added different concentrations of AA to GA solution. Regarding GA solution treated with more than 200 mg/L AA, color changes were not recognized by the naked eye before or after the reaction. We then conducted a spectrophotometric analysis in the visible region (400–800 nm). The spectrum of GA solution treated with more than 200 mg/L AA showed no peak at approximately 500 nm after the addition of NaOH with NaOCl·5H_2_O (Fig 6).

## Discussion

In the present study, we observed changes in the color, absorption spectra, and mass spectra of GA solution after the addition of NaOH with NaOCl·5H_2_O. GA solution turned dark brown in color and showed characteristic spectra. The results obtained will contribute to the development of a novel GA detection method using a spectrophotometer.

The medicinal properties of GA have been investigated widely and it was recently shown to be an effective radical scavenger, antioxidant, inhibitor of growth factors, and anti-carcinogenic substance [5–8]. Regarding pharmacokinetic and metabolic studies, GA was mainly measured by chromatography because of the difficulties associated with detecting GA and differentiating it from analogous compounds, such as ASA and other ASA metabolites, using other methods [9–11]. However, current methods for the measurement of GA require expensive analytical instruments and a complex method.

GA, a metabolite of ASA, is often present in the same sample with ASA and its metabolites, such as SA, in pharmacokinetic, pharmacodynamic, and clinical studies. Therefore, we measured the absorption spectra of ASA and SA solutions after the addition of NaOH with NaOCl·5H_2_O to examine the specificity of the characteristic GA peak. As shown in Fig 2, the absorption curves of the ASA and SA solutions did not show a color change or have any peak regardless of whether NaOH with NaOCl·5H_2_O was added. A liquid mixture of GA, ASA, and SA also showed a color change and characteristic GA peak at approximately 500 nm after the incubation with NaOH with NaOCl·5H_2_O (Fig 2). These results suggest that our simple method will enable many researchers and laboratory technicians to detect GA in samples from patients who take ASA as an anticoagulant or to control pain, inflammation, and fever. Since spectrophotometers are used at many research institutes and clinical laboratories and its operation is very simple and fast, GA may be easily detected using our method. Furthermore, it is simple to automate our method because the spectrophotometric method is installed in many automated biochemical analyzers.

The pharmacokinetics of ASA vary depending on the dose administered, age, race, and other internal and external factors [11–16]. A previous study reported that GA was associated with the toxic effects caused by the administration of ASA [17]. Our novel results on the detection of GA may contribute to the application of a safety monitoring system in order to prevent the side effects associated with ASA and its dosing period.

GA was previously shown to exert anti-rheumatic effects and the administration of gentisate reduced pain, swelling, and heat in joints with an accompanying decrease in temperature to normal levels [18]. Consden and Stanier reported that a serum sample from a rheumatic fever patient, who was treated with 22.5 g of GA over 12 hours in five equal doses at 3-hour intervals, contained 306 mg/L of GA [19]. Moreover, Roseman and Dorfman found that the urine of 5 adults, who ingested sodium gantisate in a single dose of 37 mg/kg (1.8 g– 3.7 g), contained between 196 and 714 mg of GA 2–4 hours after the ingestion of sodium gentisate [20]. Based on these findings, we estimated the detection ranges (Fig 3) and linear trends (S1 Fig) of our spectrophotometric method. GA solutions containing more than 60 mg/L showed peaks at approximately 500 nm detected by the spectrophotometer (Fig 3A) and absorbance at 500 nm showed a linear trend from 60 to 120 mg/L (Fig 3B, S1 Fig). These results indicate that our spectrophotometric method may be useful for the detection of GA after its administration.

A spectrophotometric method was previously reported for the detection of GA by measuring the ultraviolet (UV) region and GA solution showed a peak at approximately 320 nm [21]. In the present study, we measured the absorbance spectra of GA after the addition of NaOH with NaOCl·5H_2_O in the visible light region between 400 and 800 nm. We compared measurements of the absorbance spectra of GA in the UV region [21] with that of GA in the visible light region (our method) (S2 Fig, Fig 3). Although GA solutions containing between 2 and 120 mg/L showed an absorbance peak at approximately 320 nm detected by the spectrophotometer, absorbance peaks at approximately 320 nm of GA solutions containing more than 130 mg/L were not stable and accurate because these absorbance values were higher than the upper measurement limit (absorbance value of 3.0) of the spectrophotometer (S2A Fig). Furthermore, the absorbance at 320 nm of GA solutions showed linearity between 2 and 120 mg/L (S2B Fig). Absorbance peaks at approximately 500 nm of GA solutions containing more than 60 mg/L were more stable than measurements in the UV region (Fig 3A), and the absorbance at 500 nm of GA solutions showed a linear trend between 60 and 120 mg/L (S1 Fig). However, the absorbance at 500 nm of GA solutions showed non-linearity between 130 and 1000 mg/L (Fig 3B). Although the absorbance value at 500 nm of GA from 130 to 800 mg/L gradually increased, GA solutions containing more than 800 mg/L showed the decrease in absorbance (Fig 3B). These changes of the absorbance value between 130 and 1000 mg/L may be attributed to high concentration of GA for our spectrophotometric method. The oxidation reaction of GA containing more than 130 mg/L after the addition of NaOH with NaOCl·5H_2_O affected the absorption measurements at 500 nm based on the Beer-Lambert law, and the amount of light passing in high concentration was more reduced than that of low concentrations. Accordingly, the absorbance plot of GA solutions containing more than 130 mg/L showed spectral deviations from linear behavior between 60 and 120 mg/L (Fig 3B, S1 Fig). We incubated GA solutions only for 5 min after the addition of 1mol/L NaOH with NaOCl·5H_2_O at 1:50 (w/w) and were unable to establish the optimal conditions to quantify the wide range from low to high concentrations of GA in the present study. Therefore, in order to develop a quantitative method for GA, further examinations of reaction conditions to oxidize GA solutions containing more than 130 mg/L are required for the verification of a number of conditions, including the optimal incubation time and concentrations of NaOH with NaOCl·5H_2_O. We also measured the absorbance spectra of ASA, SA, GA, or a mixture solution and compared absorbance spectra in the UV region with those in the visible light region (S2 Fig, Fig 2). ASA, SA, GA, and the mixture solution (ASA, SA, and GA) showed absorbance peaks at 294, 296.5, 320, and 317 nm, respectively (S2C Fig). Based on these results, it is difficult to detect GA in samples containing ASA and SA because ASA, SA, GA, or a mixture solution showed absorbance peaks ranging between 294 and 320 nm in the UV region (S2C Fig). In contrast, as shown in Fig 2, neither ASA nor SA solutions showed color changes (Fig 2B) or absorbance peaks (Fig 2C) after the addition of NaOH with NaOCl·5H_2_O, while GA and the mixture solutions showed a color change and absorbance peak at approximately 500 nm in the visible light region (Fig 2B and 2C). These results indicate that GA is detectable in samples containing ASA, SA, and GA by measuring absorbance spectra in the visible light region after the addition of NaOH with NaOCl·5H_2_O. Based on these comparisons, our spectrophotometric method for the detection of GA by measuring the visible light region after the addition of NaOH with NaOCl·5H_2_O showed a more specific reaction than that of the UV region.

We previously analyzed the oxidation reaction of HGA to BQA after the addition of NaOH or NaOH with NaOCl·5H_2_O, which is a strong oxidant, by LC/TOF-MS and NMR spectrometry [3]. The oxidation of HGA to BQA accompanied by color changes is caused by the addition of NaOH because oxygen consumption by HGA increases at an alkaline pH [22, 23]. As shown in Fig 4, GA also showed color changes following the addition of NaOH. Since GA is a divalent phenolic acid with a similar structure and chemical properties to HGA (Fig 1), the color reaction of GA solution appears to reflect the oxidation reaction of GA caused by the addition of alkaline solution. Furthermore, we previously reported that the addition of NaOH with NaOCl·5H_2_O accelerated the oxidation of HGA to BQA [3]. As shown in Fig 4, GA solution became darker following the addition of NaOH with NaOCl·5H_2_O than with the addition of NaOH. This similar chemical property indicates that the oxidation of GA is accelerated by the addition of a combination of NaOCl·5H_2_O and NaOH. Regarding the oxidation reaction of GA, its conversion to the corresponding quinoid form, carboxybenzoquinone, has been reported [24, 25]. Furthermore, the reaction has been shown to proceed further and form dark brown compounds, characterized as the quinhydrone complex containing carboxybenzoquinone, by any oxidant [24]. We conducted a spectral analysis using LC/TOF-MS to clarify the color reaction process of GA following the addition of NaOH and NaOCl·5H_2_O (Fig 5). GA solution after the addition of NaOH or NaOH with NaOCl·5H_2_O showed a new peak at m/z 123 (Fig 5B and 5C). This peak may originate from the GA oxidant. Although we did not detect carboxybenzoquinone, the corresponding quinoid form of GA, these results indicate that the oxidation reaction of GA proceeds further and forms other compounds following the addition of a combination of NaOH and NaOCl·5H_2_O.

A previous study reported that the color of GA in urine was indistinguishable from that of alkaptonuric urine containing HGA because GA and HGA both turn dark brown in urine [26]. A recent study reported a method to identify phenolic compounds with similar structures, such as GA and HGA, using ultra-performance liquid chromatography with electrospray ionization coupled to tandem mass spectrometry (UPLC-ESI-MS/MS) [27]. However, this UPLC-ESI-MS/MS system is very expensive, time-consuming, and difficult to operate. Therefore, we compared the oxidation reaction of GA with that of HGA after the addition of NaOH with NaOCl·5H_2_O (S3 Fig) using our spectrophotometric method. GA and HGA solutions after the incubation with NaOH with NaOCl·5H_2_O both turned the same dark brown color (S3A Fig). However, the absorption curve of HGA solution showed peaks at 406 and 430 nm, while that of GA showed a peak at approximately 500 nm after the addition of NaOH with NaOCl·5H_2_O (S3B Fig). These results indicate that the spectrophotometric method using NaOH with NaOCl·5H_2_O enabled the simple, quick, and cost-effective distinction between GA and HGA without expensive analytical instruments and a complex method.

We previously reported that alkaptonuric urine and HGA solution treated with AA showed no peaks at 406 or 430 nm following the addition of NaOH with NaOCl·5H_2_O because the oxidation reaction of HGA was inhibited by the antioxidant effects of AA [3]. In order to confirm the influence of an antioxidant on the color reaction of GA, we investigated whether the characteristic peak at approximately 500 nm appeared after the addition of NaOH with NaOCl·5H_2_O. The spectra of GA solution containing more than 200 mg/L AA showed no peaks at approximately 500 nm following the addition of NaOH with NaOCl·5H_2_O (Fig 6). The inhibition of the color reaction of GA in the presence of a certain amount of the antioxidant also supports our suggestion that GA oxides are involved in the color reaction caused by NaOH with NaOCl·5H_2_O. Therefore, samples containing a high dose of AA may yield false negative results in spite of the addition of NaOH with the oxidant NaOCl·5H_2_O.

In summary, we herein demonstrated that GA solution showed characteristic absorption spectra through an oxidation reaction following the addition of NaOH with NaOCl·5H_2_O. Moreover, we confirmed that this characteristic peak at approximately 500 nm was specific to GA oxidation and distinguishable from ASA and SA. These results suggest that our spectrophotometric method will enable many researchers and laboratory technicians to detect GA in a sample solution without expensive analytical instruments or a complex method.

## Supporting information

S1 Fig Absorbance at 500 nm of GA with different concentrations. Absorbance at 500nm of GA from 60 to 120 mg/L after the addition of NaOH with NaOCl·5H_2_O. Results are the mean ± S.D. of three experiments.
(PDF)

S2 Fig Absorption spectra of GA solution in the UV region. (a) Absorption spectra of GA from 2 to 150 mg/L. (b) Absorbance at 320 nm of GA from 2 to 120 mg/L. Results are the mean ± S.D. of three experiments. (c) Absorption spectra of 50 mg/L ASA, 50 mg/L SA, 50 mg/L GA, and a mixture of GA, ASA, and SA (the final concentration of each was 50mg/L) in the UV region.
(PDF)

S3 Fig Color changes in and absorption spectra of GA and HGA. (a) 400 mg/L GA (I) and 400 mg/L HGA (II) after the addition of NaOH with NaOCl·5H_2_O. (b) Absorption spectra of 400 mg/L GA and 400 mg/L HGA after the addition of NaOH with NaOCl·5H_2_O.
(PDF)
