# Supplementary material for: Novel absorbance peak of gentisic acid following the oxidation reaction
Source: PLoS One. 2020 Apr 29;15(4):e0232263. doi: 10.1371/journal.pone.0232263 (PMC7190133; doi:10.1371/journal.pone.0232263)
Supplement: S1 Fig — Absorbance at 500nm of GA from 60 to 120 mg/L after the addition of NaOH with NaOCl·5H2O. Results are the mean ± S.D. of three experiments. (PDF) [file pone.0232263.s001.pdf]

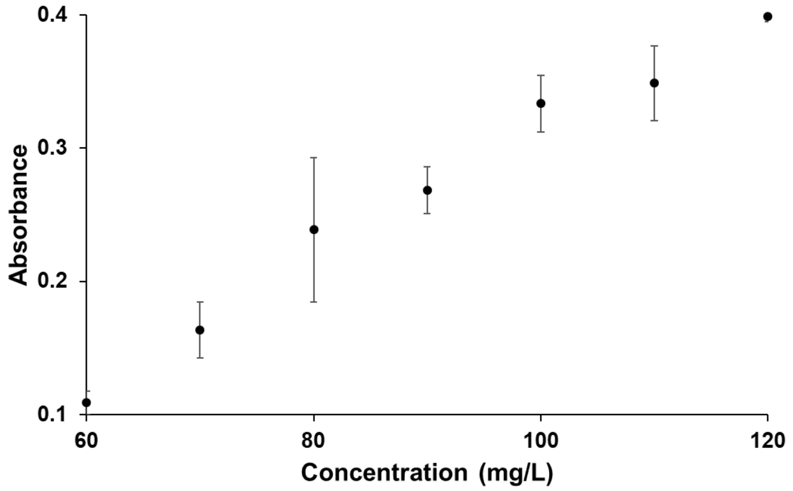

S1 Fig. Absorbance at 500 nm of GA with different concentrations.

Absorbance at 500nm of GA from 60 to 120 mg/L after the addition of NaOH with $NaOCl{\cdot}5H_2O$. Results are the mean ± S.D. of three experiments.
