# Supplementary material for: Novel absorbance peak of gentisic acid following the oxidation reaction
Source: PLoS One. 2020 Apr 29;15(4):e0232263. doi: 10.1371/journal.pone.0232263 (PMC7190133; doi:10.1371/journal.pone.0232263)
Supplement: S2 Fig — (a) Absorption spectra of GA from 2 to 150 mg/L. (b) Absorbance at 320 nm of GA from 2 to 120 mg/L. Results are the mean ± S.D. of three experiments. (c) Absorption spectra of 50 mg/L ASA, 50 mg/L SA, 50 mg/L GA, and a mixture of GA, ASA, and SA (the final concentration of each was 50mg/L) in the UV region. (PDF) [file pone.0232263.s002.pdf]

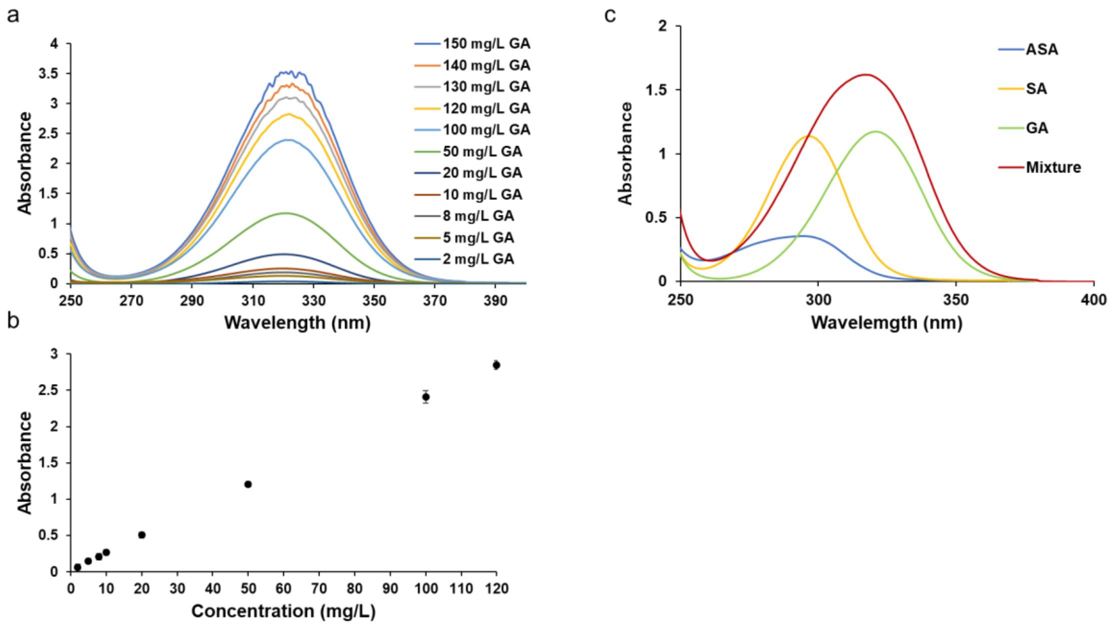

S2 Fig. Absorption spectra of GA solution in the UV region.

(a) Absorption spectra of GA from 2 to 150 mg/L. (b) Absorbance at 320 nm of GA from 2 to 120 mg/L. Results are the mean ± S.D. of three experiments. (c) Absorption spectra of 50 mg/L ASA, 50 mg/L SA, 50 mg/L GA, and a mixture of GA, ASA, and SA (the final concentration of each was 50mg/L) in the UV region.
