# Supplementary material for: Novel absorbance peak of gentisic acid following the oxidation reaction
Source: PLoS One. 2020 Apr 29;15(4):e0232263. doi: 10.1371/journal.pone.0232263 (PMC7190133; doi:10.1371/journal.pone.0232263)
Supplement: S3 Fig — (a) 400 mg/L GA (I) and 400 mg/L HGA (II) after the addition of NaOH with NaOCl·5H2O. (b) Absorption spectra of 400 mg/L GA and 400 mg/L HGA after the addition of NaOH with NaOCl·5H2O. (PDF) [file pone.0232263.s003.pdf]

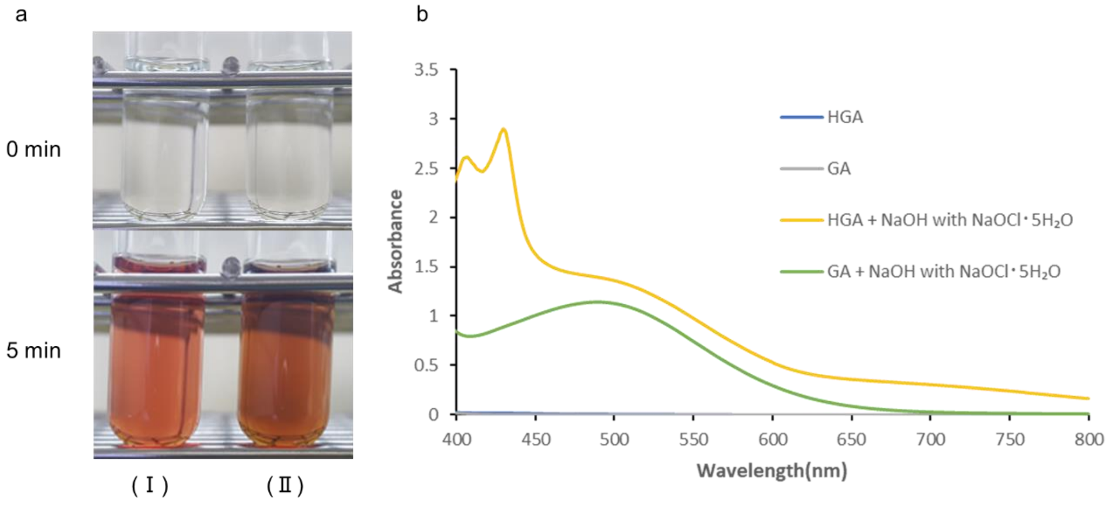

S3 Fig. Color changes in and absorption spectra of GA and HGA.

(a) 400 mg/L GA (I) and 400 mg/L HGA (II) after the addition of NaOH with $NaOCl \cdot 5H_2O$. (b) Absorption spectra of 400 mg/L GA and 400 mg/L HGA after the addition of NaOH with $NaOCl \cdot 5H_2O$.
